# Generation of Chimeric “ABS Nanohemostat” Complex and Comparing Its Histomorphological *In Vivo* Effects to the Traditional Ankaferd Hemostat in Controlled Experimental Partial Nephrectomy Model

**DOI:** 10.1155/2013/949460

**Published:** 2013-02-20

**Authors:** Emre Huri, Yavuz Beyazit, Rashad Mammadov, Sila Toksoz, Ayse B. Tekinay, Mustafa O. Guler, Huseyin Ustun, Murat Kekilli, Mumtaz Dadali, Tugrul Celik, Müzeyyen Astarci, Ibrahim C. Haznedaroglu

**Affiliations:** ^1^Department of Urology, Ankara Training and Research Hospital, Ulucanlar, 06340 Ankara, Turkey; ^2^Department of Gastroenterology, Turkiye Yuksek Ihtisas Training and Research Hospital, Sihhiye, 06100 Ankara, Turkey; ^3^Institute of Materials Science and Nanotechnology, National Nanotechnology Research Center (UNAM), Bilkent University, 06800 Ankara, Turkey; ^4^Department of Pathology, Ankara Training and Research Hospital, Ulucanlar, 06340 Ankara, Turkey; ^5^Department of Biochemistry, Fatih University Medical School, 06560 Ankara, Turkey; ^6^Department of Hematology, Hacettepe University Medical School, Sihhiye, 06100 Ankara, Turkey

## Abstract

*Purpose*. Using the classical Ankaferd Blood Stopper (ABS) solution to create active hemostasis during partial nephrectomy (PN) may not be so effective due to insufficient contact surface between the ABS hemostatic liquid agent and the bleeding area. In order to broaden the contact surface, we generated a chimeric hemostatic agent, ABS nanohemostat, via combining a self-assembling peptide amphiphile molecule with the traditional Ankaferd hemostat. *Materials and Methods*. In order to generate ABS nanohemostat, a positively charged Peptide Amphiphile (PA) molecule was synthesized by using solid phase peptide synthesis. For animal experiments, 24 Wistar rats were divided into the following 4 groups: Group 1: control; Group 2: conventional PN with only 0.5 ml Ankaferd hemostat; Group 3: conventional PN with ABS + peptide gel; Group 4: conventional PN with only 0.5 ml peptide solution. *Results*. Mean warm ischemia times (WITs) were 232.8  ±  56.3, 65.6 ± 11.4, 75.5 ± 17.2, and 58.1 ± 17.6 seconds in Group 1 to Group 4, respectively. Fibrosis was not different among the groups, while inflammation was detected to be significantly different in G3 and G4. *Conclusions*. ABS nanohemostat has comparable hemostatic efficacy to the traditional Ankaferd hemostat in the partial nephrectomy experimental model. Elucidation of the cellular and tissue effects of this chimeric compound may establish a catalytic spark and open new avenues for novel experimental and clinical studies in the battlefield of hemostasis.

## 1. Introduction

The use of nanomaterials in medicine involves the applications of nanoparticles and manufactured nanosystems to provide regeneration at the cellular and tissue levels [1]. Several nanomaterials have been designed to serve as drug delivery systems. They encapsulate therapeutic agents and typically carry multiple targeting motifs such as hemostasis [1, 2]. For instance, Ellis-Behnke and coworkers [2] introduced a unique nanomedicinal method to stop bleeding using a self-assembling peptide that establishes a nanofiber barrier and incorporates it into the surrounding tissue to form an extracellular matrix [1, 2].

 Biomaterials used as tissue engineering scaffolds have specific physical properties and might form fibrous networks similar to collagenous extracellular matrix. They also can be programmed to carry chemical and physical cues to provide bioactivity for cell-materials interactions. In the search for more improved bioactive materials for tissue engineering purposes, peptide amphiphile (PA) molecules are good candidates to bring scaffold properties and bioactivity together [3, 4]. Hydrophobic region of the PA is composed of fatty acids, which are packed against aqueous medium inducing self-assembly of the molecule to form cylindrical nanofibers. Charged amino acids on hydrophilic peptide region of the PA molecules are responsible for triggering self-assembly upon isolation of charges [5]. Neutralization of charge density on amino acids causes aggregation of PA molecules and formation of nanofibrous networks at microscale. Encapsulation of water by these nanofiber networks results in constitution of self-supporting gel at macroscale [6]. Peptide part of the PA molecules can be synthesized in a way to include bioactive epitopes or biofunctional chemical groups, which are eventually presented to cells on nanofibers. PA gels carrying these types of functionalities have been studied thoroughly and proven to be active in terms of inducing angiogenesis, neuronal regeneration, and cartilage and bone tissue formation [7–10]. Charge neutralization mechanism also allows us to use bioactive molecules with high negative or positive charge density, such as heparin, DNA, or oligonucleotides for inducing gel formation, while exploiting their bioactivity [6, 11, 12].

The use of medicinal plants as remedies for numerous disorders has formed the basis of current medicinal approach. Various plants are used ethnomedicinally for prevention of excessive bleeding and as wound dressing to staunch blood flow [13]. Ankaferd hemostat, a topical hemostatic agent of plant origin, has recently been registered for the management of clinical hemorrhages when the conventional methods to control bleeding by ligature and/or hemostatic measures are ineffective [14–16]. Ankaferd Blood stopper (ABS) includes standardized preparation of the plants *Thymus vulgaris*, *Glycyrrhiza glabra*, *Vitis vinifera*, *Alpinia officinarum*, and *Urtica dioica* [17, 18].

ABS provides vital erythroid aggregation covering the entire physiological hemostatic process via a unique protein network depending primarily on the interactions between ABS and blood proteins, particularly with fibrinogen gamma and prothrombin [16, 19–22]. Vital erythroid aggregation takes place with the spectrin and ankyrin receptors on the surface of red blood cells (RBCs). Those RBC proteins and the required ATP bioenergy are included in the ABS protein library [23, 24]. On the other hand, controlled clinical trials indicated the safety and efficacy of topical Ankaferd hemostat in distinct clinical backgrounds [19, 25–31]. 

In this work, we present a chimeric hemostatic agent, ABS Nanohemostat, via combining a self-assembling PA molecule with the traditional Ankaferd hemostat. The first step of our research was the synthesis of the specific self-assembling peptide molecules capable of being a part of the combined ABS Nanohemostat compound. The second step was the assembly of the peptide nanofibers and ABS to generate the ABS Nanohemostat. The third step of our study was testing the *in vivo* hemostatic effects of ABS Nanohemostat in comparison with the traditional Ankaferd in a previously established and published controlled surgical experimental trial in kidney tissue [32]. The ultimate aim of the present study is, therefore, to test the efficacy of ABS Nanohemostat formed by combination of a self-assembling PA molecule and Ankaferd hemostat for controlling surgical bleeding due to renal injury during partial nephrectomy (PN) and to compare its hemostatic effects to the traditional surgical and Ankaferd hemostat groups. 

## 2. Materials and Methods

### 2.1. Nanomedicinal Approach and ABS Nanohemostat

#### 2.1.1. Materials

9-Fluorenylmethoxycarbonyl (Fmoc), ter. Butoxycarbonyl (Boc) protected amino acids, Rink Amide MBHA resin, and 2-(1H-Benzotriazol-1-yl)-1,1,3,3-tetramethyluronium hexafluorophosphate (HBTU) were purchased from NovaBiochem or ABCR. The other chemicals were purchased from Fisher, Merck, Alfa Aesar, or Aldrich and used as provided.

#### 2.1.2. Peptide Synthesis

**Figure 1:**
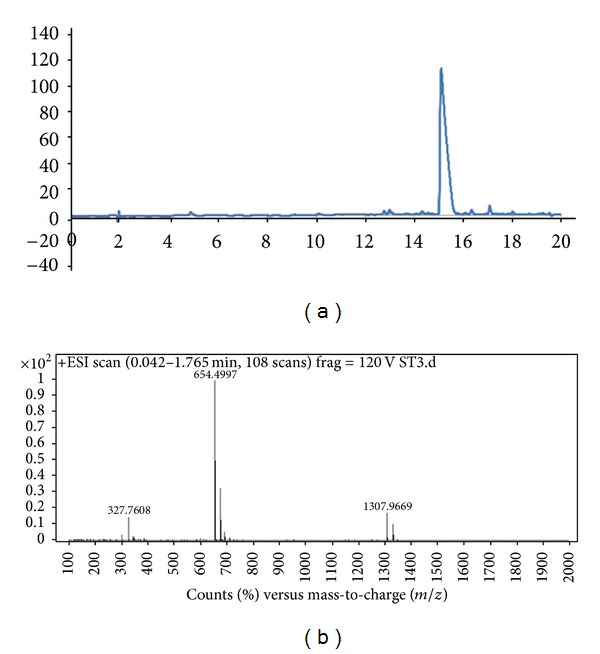
Liquid chromatography (a) and mass spectrometry (b) characterization of the peptide amphiphile (PA) molecule.

Peptides were constructed on Rink Amide MBHA resin. Amino acid couplings were done with 2 equivalents of Fmoc protected amino acid, 1.95 equivalents HBTU, and 3 equivalents of N,N-diisopropylethylamine (DIEA) for 2 hours. Fmoc removal was performed with 20% Piperidine/Dimethylformamide (DMF) solution for 20 min. Cleavage of the peptides from the resin was carried out with a mixture of TFA : TIS : H_2_O in ratio of 95 : 2.5 : 2.5 for 2 h. Excess TFA was removed by rotary evaporation. The remaining viscous peptide solution was triturated with ice-cold ether, and the resulting white product was dried under vacuum. PA molecules were characterized by liquid chromatography-mass spectrometry (LC-MS) (Figure 1). Mass spectrum was obtained with Agilent LC-MS equipped with Agilent Zorbax Extend-C18 2.1 × 50 mm column. A gradient of (a) water (0.1% formic acid) and (b) acetonitrile (0.1% formic acid) was used. 

#### 2.1.3. Peptide Amphiphile Molecule Design

**Figure 2:**
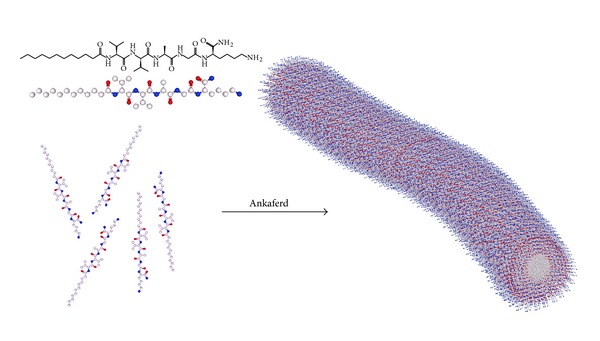
Schematic representation of ABS Nanohemostat formation by self-assembly of peptide amphiphile molecules into nanofibers upon addition of Ankaferd solution.

The PA was synthesized by Fmoc Solid Phase Peptide Synthesis (SPPS) method. It is composed of a lauryl (C12) group, hydrophobic region of the PA, and a peptide sequence. VVAG peptide sequence is used as *β*-sheet inducer that causes nanofiber formation, while the lysine (K) residue is protonated at physiological pH and increases solvation of PA molecule in aqueous solution at pH 7.  Figure 2 shows both the chemical structure of the PA (Lauryl-VVAGK-Am) and the schematic representation and ABS Nanohemostat formation.

#### 2.1.4. Circular Dichroism (CD)

JASCO J815 CD spectropolarimeter was used at room temperature. 1 × 10^−4^ M peptide solutions were measured from 300 nm to 190 nm, data interval and data pitch being 0.1 nm and scanning speed being 100 nm/min, all measurements with three accumulations. Digital integration time (DIT) was selected as 1 sec, band width as 1 nm, and the sensitivity was standard.

#### 2.1.5. Rheology

Oscillatory rheology measurements were performed with Anton Paar Physica RM301 Rheometer operating with a 25 mm parallel plate configuration at 25°C. Each sample of 100 *μ*L total volume with a final peptide concentration of 1 wt% was carefully loaded on the center of the lower plate and incubated for 15 min before measuring. After equilibration, the upper plate was lowered to a gap distance of 0.5mm. Storage moduli (G′) and loss moduli (G′′) values were scanned from 100 rad/s to 0.1 rad/s of angular frequency, with a 0.5% shear strain.

#### 2.1.6. Scanning Electron Microscopy (SEM) Imaging

**Figure 3:**
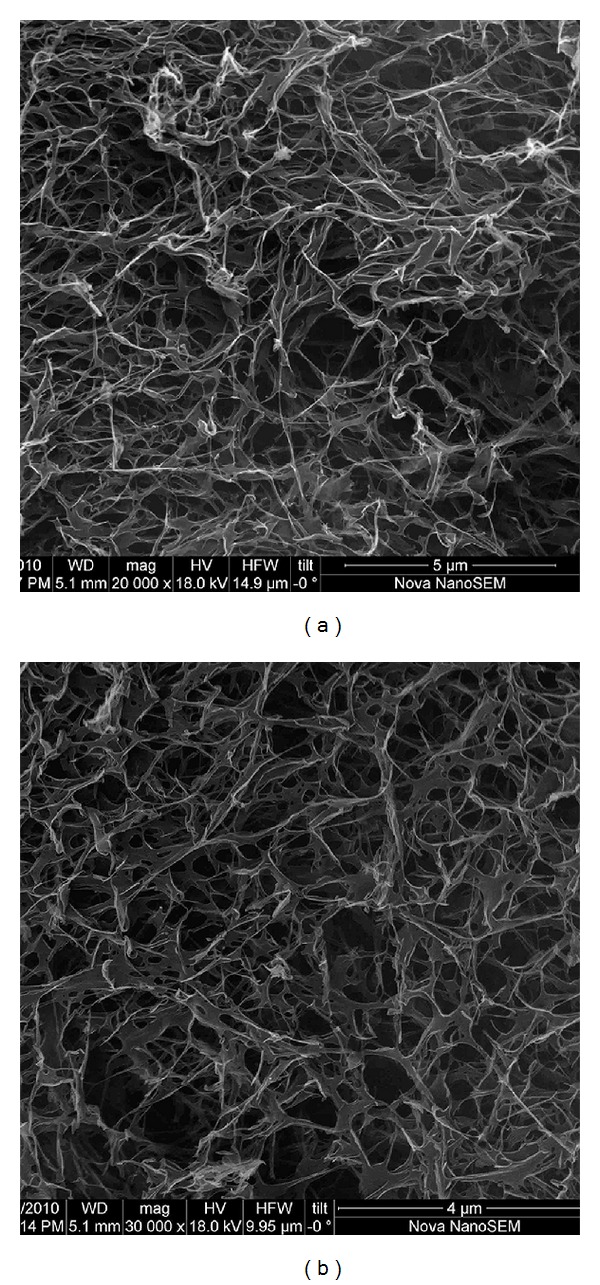
Scanning Electron Microscopy images of peptide amphiphile (PA) gel with Ankaferd (a) or alone (b) at pH 10.

SEM experiments were performed with FEI Nova NanoSEM 230, using the ETD detector at low vacuum mode with 30 keV beam energy. Small amounts of gels with a final peptide concentration of 1% were put on a metal mesh, dried at critical point (1072 Psi, 31°C) with Tousimis Autosamdri-815 B Series C critical point dryer and coated with 6 nm Au-Pd.  Figure 3 shows SEM images of PA gel with and without Ankaferd at pH 10.

#### 2.1.7. *In Vivo* Application of PA and Ankaferd Gel

An amount of 250 *μ*L of PA was mixed with 250 *μ*L ABS solution on glass slide and incubated at room temperature for 30 min for self-supporting gel formation. Due to anionic molecules in Ankaferd solution, self-supporting gel was formed immediately after mixing. This gel was applied directly onto wound area.

### 2.2. Surgical Approach and ABS Nanohemostat

#### 2.2.1. Animals

All animal experimentations described in this paper were carried out in accordance with national guidelines for the use and care of  laboratory animals and were approved by the local animal review and ethics committee. All procedures were in full compliance with Turkish Law 6343/2, Veterinary Medicine Deontology Regulation 6.7.26, and with the Helsinki Declaration of World Medical Association recommendations on animal studies. The animals were obtained from the center of medical experimental research of Ankara Training and Research Hospital. The rats were housed in stainless steel cages in an animal room maintained at a temperature of 22–24°C with 12-hour light/dark periods. All were fed with the same amount of laboratory pellet diet and with water supplied *ad libitum* for a minimum of 5 days before procedure.

#### 2.2.2. Rat Model of Partial Nephrectomy

A total of 24 Wistar rats weighing 200 to 300 g were divided into 4 groups of 6 each and underwent PN. One surgeon with an assistant performed all the surgical procedures. All operations were performed under total anesthesia with injection of 50 mg/kg intramuscular ketamine hydrochloride. After sterile preparation and draping, a midline incision was made on the abdomen. For each rat, renal artery and vein were revealed by hilar vascular dissection. Subsequently, renal artery and vein were clamped with Rommel vascular clamp. The lower third of the left kidney was resected in guillotine fashion with a single stroke of an amputating knife. Four different hemostatic techniques were applied to the groups.

 (i) Group 1 (G1) is the left PN with hilar vascular control including intracorporeal suturing of the renal parenchyma and collecting duct (control group).
 (ii) Group 2 (G2) is the conventional PN with only 0.5 mL traditional Ankaferd hemostat (ABS) application without suturing.
 (iii) Group 3 (G3) is the conventional PN with ABS (0.25 mL) + peptide (0.25 mL) gel (ABS Nanohemostat) mixture application with no suturing.
 (iv) Group 4 (G4) is the conventional PN with only 0.5 mL peptide solution application.

Two objective parameters were recorded during the surgical procedure: warm ischemia time (WIT) and amount of bleeding (AOB). The unit of WIT was the “second,” while the AOB was measured with the bleeding area (cm^2^) onto the sponges. The abdominal incision was afterwards closed with surgical sutures. All the rats were allowed to feed and drink water for the following 4 weeks. After that, each rat was sacrificed, and total nephrectomy was performed for histopathological examination.

#### 2.2.3. The Hemostatic Methods during PN

**Figure 4:**
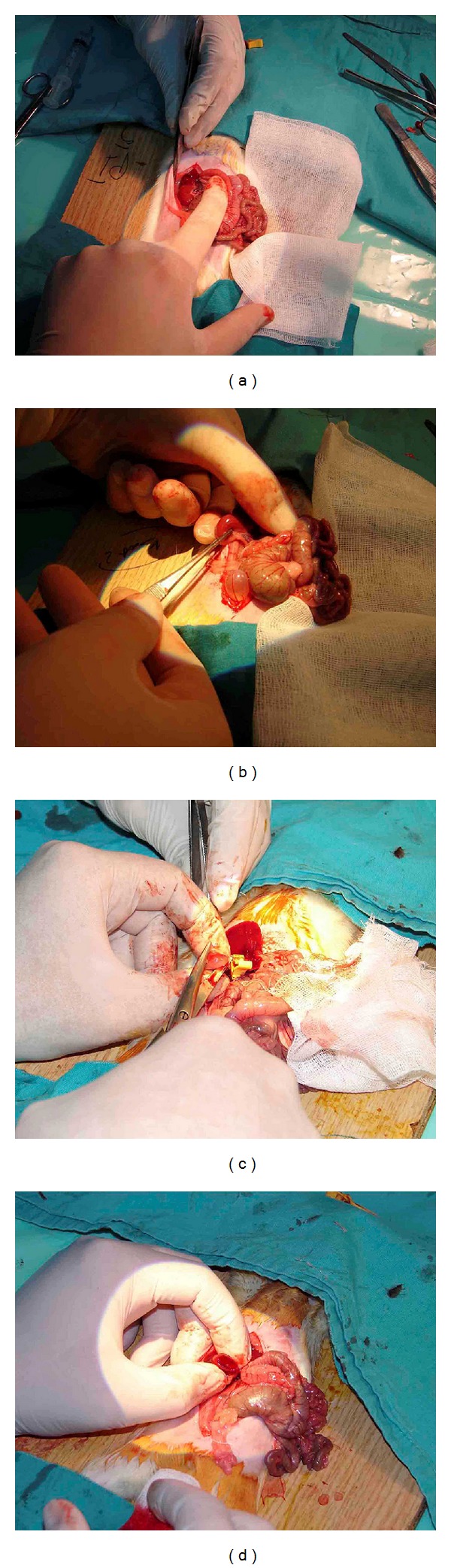
(a) and (b) Traditional partial nephrectomy model with suture on resected area dissection of kidney. (c) and (d) Transection of kidney and application of ABS.

Each hemostatic method was used during the period of warm ischemia (WI). WI started with clamping the renal artery and vein and finished with taking the clamp out. In G1, traditional hemostasis method was used as compression onto the renal excised area and suturing the renal vessels and collecting duct with absorbable sutures (Figures 4(a) and 4(b)). In G2, 2 mL of ABS was dropped to the amputated renal margin steadily until bleeding stopped (Figures 4(c) and 4(d)). In G3, ABS (2 mL) + Nanopeptide gel mixture (ABS Nanohemostat) was applied onto the injured area. And in G4, only nanopeptide gel was used to control bleeding. Other hemostatic methods including sponges, Surgicel, electrocautery, and any other sources were not used to control bleeding in the present study. 

#### 2.2.4. Histopathological Evaluation

Light microscopic sections were reviewed by a pathologist blinded to the treatment groups. Coronal 1 to 2 mm thick slices of the kidney were fixed in 4% formaldehyde and embedded in paraffin, and 3 to 4 *μ*m thick tissue sections were stained with hematoxylin and eosin. Sections were examined for several parameters including erythroid aggregation, fibrosis, calcification, inflammatory cells, and siderophages. For each case, a global estimation of parenchymal damage was done and semiquantitatively graded on a scale in which 0 was no abnormality, and 1+, 2+, and 3+ represented mild, moderate, and severe abnormalities, respectively.

#### 2.2.5. Statistical Analyses

Data analysis was performed using IBM Statistical Package for the Social Sciences (SPSS) statistics software. Continuous variables were tested for normality by the Kolmogorov-Smirnov test. Values were presented as median and range because all numeric data were nonnormally distributed. Comparisons of numerical variables among four groups were initially performed using the Kruskal-Wallis test and Mann-Whitney *U*-test with the Bonferroni correction used for post hoc analysis to compare the groups separately. A *P* value of <0.0083 (0.05/6) was deemed statistically significant in multiple comparisons. The percentages of histopathological features between study groups were compared using the chi-squared test. A *P* value of <0.05 was considered as statistically significant. 

## 3. Results

### 3.1. ABS Nanohemostat Gel Evaluation

**Figure 5:**
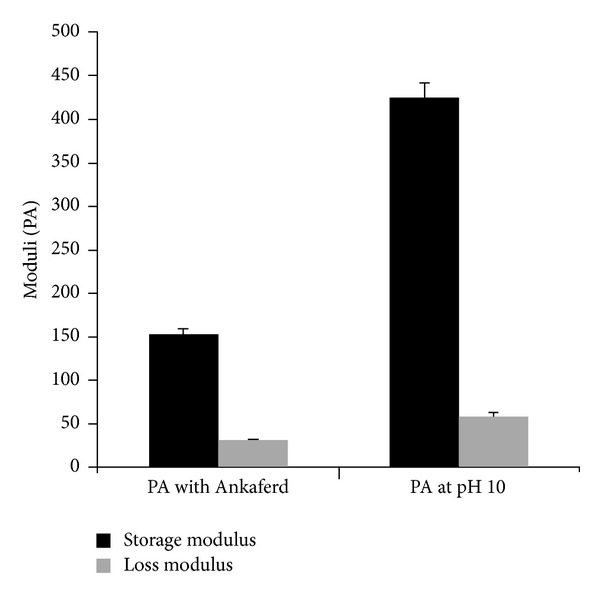
Comparison of mechanical character of PA-Ankaferd gel with PA gel formed at pH 10 by using oscillatory rheology.

 In this work, we synthesized a positively charged PA molecule as shown in Figure 2. Purity of the molecule was demonstrated by using liquid chromatography and mass spectrometry. Storage and loss moduli measurement by oscillatory rheometry was performed in order to characterize the mechanical properties of the gel. Storage moduli, showing stiffness of gel, were found to be several folds higher than loss moduli, indicating a self-supporting gel formation (Figure 5). Moreover, storage modulus value was comparable to control gel formed by elevating pH of PA solution. Circular dichroism spectroscopy was used to investigate the secondary structure of the PA and Ankaferd mixture. Characteristic *β*-sheet formation was observed in PA-Ankaferd solution, which was similar to PA solution at high pH. Morphology of the gel was examined by using SEM which showed nanofibrous network structure similar to PA gels reported before and PA samples at high pH. The pH 10 solutions for PA molecule were studied to analyze the peptide gels alone. The PA molecules were dissolved at pH 7, and they self-assembled into nanofibers forming gels upon addition of Ankaferd solution or by increasing the pH to 10.

### 3.2. Evaluation of the Study Groups

Left lower pole PN was surgically performed successfully to each rat in all groups. The kidney size and shapes were similar. Likewise, the size of the resected area was standardized for groups. The same surgical equipments were used, and Rommel clamps succeeded the warm ischemia during all surgical procedures. 

### 3.3. Perioperative and Postoperative Findings

**Figure 6:**
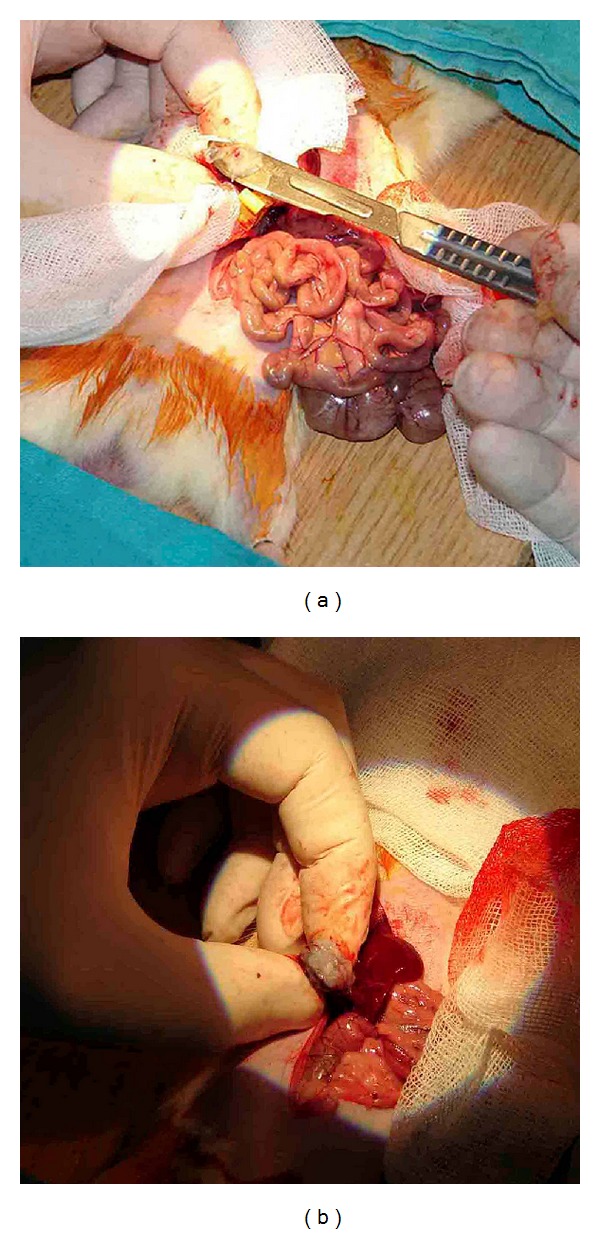
(a) and (b) Application of the ABS Nanohemostat on the resected area.

 Mean ± SD WITs were 232.8 ± 56.3, 65.6 ± 11.4, 75.5 ± 17.2, and 58.1 ± 17.6 seconds in G1 to G4, respectively. A significant difference was detected between G1 and G2, 3, and 4, while no difference was observed among the three groups; G1, G2, and G3 (Table 1). In G1, renal parenchyma injury that caused the longer WIT and repaired with primary suture was present in one rat. In G4, more amount of Nanopeptide agent was used to stop the bleeding in one rat following the warm ischemia. Apart from these two cases, no complication was observed during the operations. Application of the ABS Nanohemostat on the resected area was shown in Figure 6. Hemostasis was detected macroscopically in all rats. 

### 3.4. Histopathological Evaluation after Scarification

**Figure 7:**
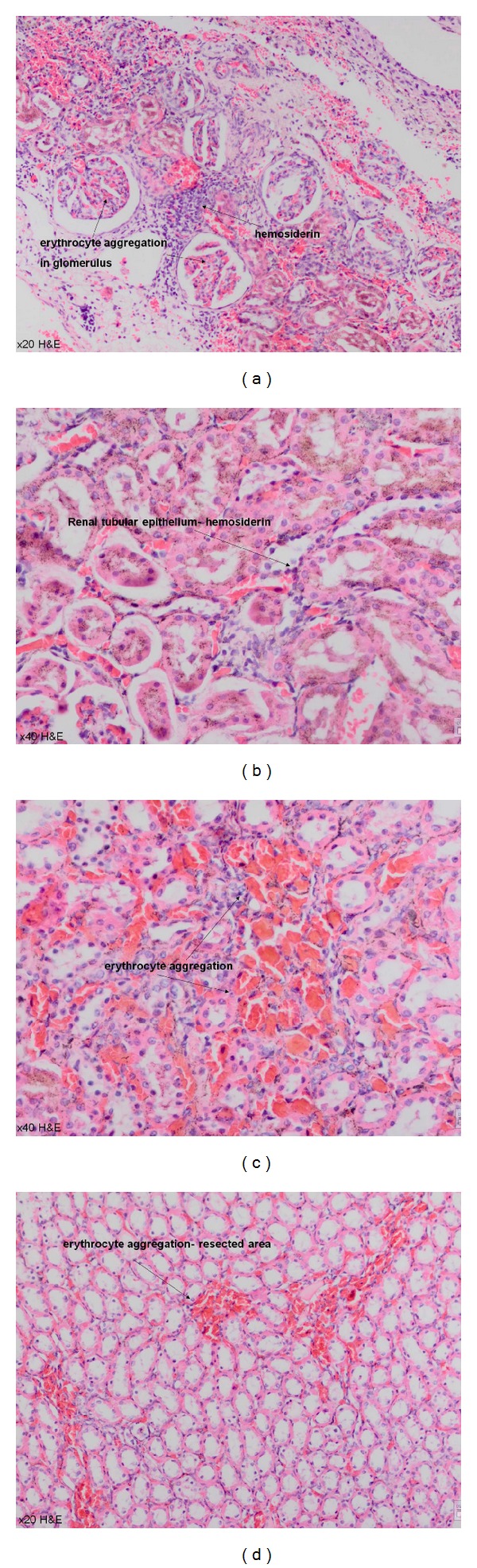
Erythrocyte aggregation in glomerular field and interstitium in ABS Nanohemostat group (20–40 xHE).

**Figure 8:**
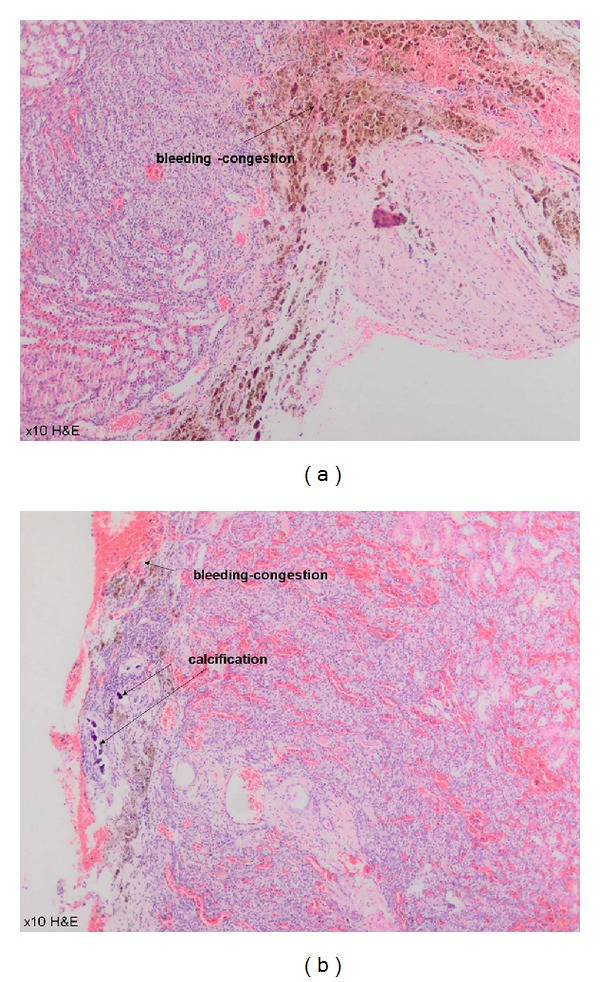
Bleeding and congestion without erythrocyte aggregation with significant calcification in Nanopeptide group (10 xHE).

All rats were sacrificed at the first month following the surgery. Each specimen was protected in formalin solution. Fibrosis was not different among the groups (*P* > 0.05), while inflammation was detected to be significantly different in G3 and G4 (*P* = 0.04). Erythrocyte aggregation, especially in glomerular field (Figure 7), was confirmed to be significantly higher in G3 (ABS Nanohemostat) compared with the other groups (*P* = 0.03). Hemosiderin was also detected in G3; however, no significant difference was detected among the groups. In G4, bleeding and congestion without erythrocyte aggregation were confirmed with significant calcification (*P* < 0.001) (Figure 8).  Table 2 shows the histopathological features of the study groups. We confirmed the significant demonstrative aggregation of red blood cells in ABS Nanohemostat group as shown in ABS group. Giant cell reaction, thyroidization, fibroblast activation, and microvascular proliferation were not detected. 

## 4. Discussion

In this study, we revealed that PA-Ankaferd gel mixture (ABS Nanohemostat) is effective as traditional Ankaferd hemostat. Moreover, this unique nanomedicine proves itself as an effective and easily applicable alternative hemostatic method which can successfully be used even in complicated hemorrhagic situations such as aggressive surgical tissue bleeding due to PN. 

Several hemostatic agents are preferred to control external and internal bleedings; yet, commercially available products are not sufficiently effective or fast acting to achieve hemostasis in extreme occasions. Ankaferd hemostat is a topical hemostatic agent of plant origin, including molecules with high density of negative and positive charges, and is proven to work as an efficient hemostatic agent [14–16]. Controlled clinical trials indicated the safety and efficacy of topical Ankaferd hemostat in distinct clinical backgrounds [19, 25–31]. In this work, we aimed to assemble a nanostructured scaffold material with hemostatic activity in the PA and Ankaferd gel formed upon mixing soluble PA molecule with Ankaferd solution (ABS Nanohemostat). Thus, we hypothesized that, while reducing blood loss, nanofiber network will serve as a scaffold for infiltrating cells to wound area, and eventually tissue regeneration rate will be enhanced.

Sustaining hemostasis in clinical hemorrhages is a challenging task and requires extensive effort to stabilize traumatic and surgical injuries. Our experimental animal model is therefore clinically relevant since bleeding is one of the most important major complications leading to the morbidity and even mortality during PN for renal masses. Providing hemostasis following the renal mass excision is the most important step of PN [32], and various hemostatic agents were used to stop bleeding during this operation [33–35]. Although the complication rates including bleeding are slightly higher than those of open radical nephrectomy, the advantage of renal preservation is more evident [32]. Likewise, laparoscopic PN, including hilar control, suture repair of the collecting system, suture ligation of blood vessels, and capsular closure, is also a valuable urological intervention. Bleeding and ischemic renal damage due to warm ischemia period are the most important complications following surgery. In order to decrease WIT and PNT, various tissue sealant and haemostatic agents have been developed to replace tissue suturing. Several agents have been investigated for their hemostatic potential in managing vascular injury and many have also been evaluated for their efficacy on repairing the collecting system injury. In our results, ABS and ABS Nanohemostat applications onto the transected kidney area provided active hemostasis in PN with significant decrease in WITs and PNTs, comparable with suture group. With more application number of ABS products, active hemostasis was also observed without hilar occlusion. However, the absence of urinoma in the groups applied with ABS may reveal the effect of ABS on collecting system repair following one month. These favorable findings lead to the active hemostasis and regular healing of transected kidney without adherence and urinoma around the kidney [32]. In our studies, we demonstrated foreign material reaction in conventional PN model but not observed in the ABS groups. The immunogenicity of the hemostatic molecules was out of this present study. However, it could be speculated that the topical ABS administration onto the sutured line might prevent foreign biological reactions (FBRs) against the suture by the way of affecting biological pathways. The anti-infective effects [36–38] of ABS represent a putative biological mode of action on the hypothetical FBR. 

The hemostatic effect of local ABS application in a patient who underwent an open PN has already been shown [34]. ABS was successfully applied to stop bleeding without suturing the renal parenchyma, which provided active hemostasis in PN on the transected kidney area [24]. The surgical and histopathological findings about PN in the present trial and our previous research [32] suggested similar results regarding the renal tissue effects of ABS. Moreover, nanotechnologically generated chimeric ABS Nanohemostat was observed to increase the duration of the contact of Ankaferd hemostatic agent with the bleeding area during PN via the controlled targeted release of the topical hemostatic agent to the tissue.

Controlled clinical studiesconducted to evaluate the effectiveness of ABS in distinct states of bleeding disorders documented the safety and efficacy of traditional Ankaferd hemostat in comparison to conventional antihemorrhagic medications. The first randomized controlled clinical study was reported by Teker et al. [39] in 49 patients with anterior epistaxis. In this study, patients were randomly grouped to receive the hemostasis technique by means of either ABS wet tampon or phenylephrine impregnated gauze tampon for anterior epistaxis control. ABS successfully treated all bleeding intensity with one application (for 5 m). ABS patients experienced fewer rebleeding rates within 7 days compared to phenylephrine patients (8.3 versus 20%, *P* < 0.05). In patients to whom ABS was applied, significant differences in effective control of anterior epistaxis were observed compared to phenylephrine [31]. Similarly, another clinical trial has also demonstrated the effectiveness of ABS in children undergoing tonsillectomy [30]. In this prospective controlled study, the success of ABS and the traditional knot-tie approach to reach hemostasis for patients undergoing tonsillectomy was evaluated. Forty-seven consecutive patients undergoing cold knife dissection tonsillectomy were studied, in all of whom ABS wet tampon was used for right side tonsil hemorrhage and knot-tie technique for left side tonsil hemorrhage. The ABS side was reported to have shorter hemostasis time after tonsil removal than the knot-tie side (3.19 ± 0.74 min versus 7.29 ± 2.33 min, *P* < 0.01) and less blood loss (1.57 ± 2.26 mL versus 14.04 ± 7.23 mL, *P* < 0.01). The author concluded that ABS is not only safe and efficient, but also it decreases intraoperative bleeding and reduces operating time compared to the traditional hemostasis methods after cold knife dissection tonsillectomy [30]. The effectiveness of ABS in cancer patients was also reported in a study by Al et al. [25]. Sixty-nine cancer patients that were admitted for port insertion to a university hospital were randomized either to take a wet compress form of ABS or regular dry sterile sponges to stop the bleeding that occurs during the clinically indicated vascular port insertion. The average time needed to stop the bleeding was 32.97 ± 29.9 s for ABS group and 123.75 ± 47.5 s for dry sponge group. ABS was proven to stop local bleeding in a shorter time, with a lower recurrence rate in comparison with the sterile sponge [25]. Additional clinical studies about antihemorrhagic efficacy of ABS on the bleeding due to adenoidectomy, [28, 40] the surgical bleeding of thyroidectomy, [41] gastrointestinal bleedings [14, 29], and dental bleedings [27] were performed with similar beneficial outcomes. Our promising experimental results about ABS Nanohemostat in this study cast novel studies to search clinically measurable end-points regarding the hemostatic efficiency of this unique chimeric nanomedicine. The findings of our present study represent a starting point to investigate clinical antihemorrhagic effects of ABS Nanohemostat.

The ability of ABS to induce formation of a protein network not only makes it an effective hemostatic agent, but also confers anti-infective, antineoplastic, and healing modulator properties to the extract [14–16]. Those pleiotropic effects were ascribed to the ABS-induced alterations in apoptosis, angiogenesis, cellular proliferation, and cellular dynamics [4–6]. In previous investigations, histopathological examination of the damaged vascular structures revealed ABS-induced red blood cell aggregates supporting the hypothesis that ABS-induced formation of the protein network with vital erythroid aggregation covers the entire physiological hemostatic process [42, 43]. We have observed the same structures in the kidney tissue in the present study. In the current study, ABS Nanohemostat has led to a more pronounced erythroid aggregation at the tissue level in comparison to the traditional Ankaferd hemostat. However, clinical antihemorrhagic efficiency of Ankaferd solution seems to be superior to the chimeric Ankaferd Nanohemostat.

## 5. Conclusions

In summary, ABS Nanohemostat has comparable hemostatic efficacy to the traditional Ankaferd hemostat in the PN experimental model. ABS Nanohemostat-induced erythroid aggregation is prominent at the kidney tissue level. These observations prompt the design of future experimental and clinical studies focusing on the antibleeding and vascular repair effects of this novel hemostatic nanomedicine. The production of ABS Nanohemostat in gel formation is also important for using the hemostatic material during the laparoscopic urologic surgery. The steady scaffold onto the resected area with ABS Nanohemostat predicts the intracorporeal use of ABS hemostat as a promising agent. Likewise, future controlled studies are needed to shed further light on the expanding spectrum of the effects of ABS compounds in hemostasis and related areas.

## Figures and Tables

**Table 1:** Comparison of warm ischemia time (WIT) and amount of bleeding (AOB) in study groups.

	WIT (sec)	AOB (cm^2^)
Group 1 (*n* = 6)	232.8 ± 56.3	7.3 ± 3.3
Group 2 (*n* = 6)	65.6 ± 11.4	5.7 ± 2.3
Group 3 (*n* = 6)	75.5 ± 17.2	5.2 ± 3.2
Group 4 (*n* = 6)	58.1 ± 17.6	16.4 ± 7.7
*P*	0.003*	0.035**

*G1 versus G2 *P* = 0.0002; G1 versus G3 *P* = 0.002; G1 versus G4 *P* = 0.002; G2 versus G3 *P* = 0.31; G2 versus G4 *P* = 0.699; G3 versus G4 *P* = 0.24.

**G1 versus G2 *P* = 0.48; G1 versus G3 *P* = 0.39; G1 versus G4 *P* = 0.035; G2 versus G3 *P* = 0.69; G2 versus G4 *P* = 0.015; G3 versus G4 *P* = 0.015.

**Table 2:** Histopathological features of study groups.

Parameters	Group 1 (%)	Group 2 (%)	Group 3 (%)	Group 4 (%)	*P*
Presence					
Fibrosis	0	1 (16.6)	3 (50)	2 (33.2)	NS
Inflammation	0	1 (16.6)	4 (66.7)	4 (66.7)	0.048*
Erythroid aggregation	2 (33.2)	1 (16.6)	5 (83.3)	5 (83.3)	0.036*
Hemosiderin	2 (33.2)	2 (33.2)	5 (83.3)	4 (66.7)	NS
Calcification	0	0	6 (100)	5 (83.3)	<0.001*
Overall (*n*)	6 (100)	6 (100)	6 (100)	6 (100)	

NS: not significant.

*Significant difference was found between G1 and G2 versus G3 versus G4.
